# ARHGAP15 in Human Breast Carcinoma: A Potent Tumor Suppressor Regulated by Androgens

**DOI:** 10.3390/ijms19030804

**Published:** 2018-03-10

**Authors:** Kiyoshi Takagi, Yasuhiro Miki, Yoshiaki Onodera, Takanori Ishida, Mika Watanabe, Hironobu Sasano, Takashi Suzuki

**Affiliations:** 1 Departments of Pathology and Histotechnology, Tohoku University Graduate School of Medicine, 2-1 Seiryo-machi, Aoba-ku, Miyagi-ken, Sendai 980-8575, Japan; t-suzuki@patholo2.med.tohoku.ac.jp; 2 Department of Disaster Obstetrics and Gynecology, International Research Institute of Disaster Science, Tohoku University, Sendai, Miyagi 980-8574, Japan; miki@patholo2.med.tohoku.ac.jp; 3 Departments of Anatomic Pathology, Tohoku University Graduate School of Medicine, Sendai, Miyagi 980-8575, Japan; golgo04@magic.odn.ne.jp (Y.O.); hsasano@patholo2.med.tohoku.ac.jp (H.S.); 4 Departments of Breast and Endocrine Surgical Oncology, Tohoku University Graduate School of Medicine, Sendai 980-8575, Japan; takanori@med.tohoku.ac.jp; 5 Department of Pathology, Tohoku University Hospital, Sendai, Miyagi 980-8574, Japan; mkawatan@patholo2.med.tohoku.ac.jp

**Keywords:** breast carcinoma, ARHGAP15, Rac1, immunohistochemistry, androgens

## Abstract

Rho GTPase activating protein 15 (ARHGAP15) is a recently identified GTPase activating protein which enhances intrinsic hydrolysis of GTP-bound Ras-related C3 botulinus toxin substrate (Rac1), resulting in inactivation of Rac1. Although a lot of studies have pointed out the pivotal roles of the Rac1 pathway in the progression of breast carcinomas, the clinical significance of ARHGAP15 has remained largely unknown in human breast carcinomas. Therefore, we immunolocalized ARHGAP15 in one hundred breast carcinoma tissues. ARHGAP15 immunoreactivity was frequently detected in the cytoplasm of carcinoma cells, and was positively correlated with that of Rac1 and androgen receptor labeling index. Furthermore, ARHGAP15 immunoreactivity was significantly correlated with decreased risk of recurrence and improved prognosis, and multivariate analyses demonstrated that ARHGAP15 immunoreactivity was an independent prognostic factor for both disease-free and breast-cancer-specific survival of the patients. In addition, exogenous overexpression of ARHGA15 suppressed cell proliferation and migration of MCF-7 cells and SK-BR-3 cells. On the other hand, *ARHGAP15* mRNA was significantly induced by dihydrotestosterone. These findings suggest that *ARHGAP15* is an androgen-induced gene and has anti-tumorigenic roles associated with the Rac1 pathway. ARHGAP15 immunoreactivity is therefore considered a potent prognostic factor in human breast carcinomas.

## 1. Introduction

Breast carcinoma is a common malignancy in women all over the world. Breast tissue is a target of sex steroids, and estrogens and androgens have important roles not only in normal breast development but in the progress of human breast carcinomas.

Ras homolog family (Rho)/Ras-related C3 botulinus toxin substrate (Rac) GTPases are a family of small G-proteins and regulate many biological processes such as cytoskeleton rearrangement and cellular adhesion, migration, proliferation, survival, and so on [1]. Moreover, accumulating studies have suggested the importance of Rho/Rac GTPases in the progress of human malignancies including breast carcinomas [2,3]. The Rho/Rac GTPase family consists of six classes: Rho (RhoA, RhoB, and RhoC), Rac (Rac1, Rac2, and Rac3), and cell division cycle 42 (Cdc42) (Cdc42, RhoQ, RhoJ, RhoV/Wnt-1 resposive Cdc42 homolog-2 (Wrch-2) and Wrch-1), Rho related BTB domain containing (RhoBTB), Rho family GTPase (Rnd), and RhoT [2,4]. Among these, RhoA, Rac1, and Cdc42 are well studied. Rho GTPases serve as molecular switches which fluctuate between the inactive GDP-bound form and the active GTP-bound form, and two types of proteins are mainly involved in the regulation of small GTPase activity: guanidine nucleotide-exchanging factors (GEFs) and guanidine nucleotide-activating proteins (GAPs). GEFs activate small GTPases by catalyzing the exchange of GDP to GTP, while GAPs promote intrinsic hydrolysis of bound GTP molecules (GTP to GDP), resulting in inactivation of small GTPases [2]. In addition, guanine nucleotide dissociation inhibitors (GDIs) bind to small GTPases, sequester them in the cytosol, and maintain them in an inactive state [5]. To date, several studies have indicated the importance of Rho/Rac GTPases as well as Rho/Rac GEFs in breast carcinomas. For example, Rac1b, a constitutively active isoform of Rac1, are overexpressed in breast carcinomas, while Rac1 and Cdc42 are associated with growth-factor-mediated proliferation, invasion, and therapeutic resistance [6,7]. Rac GEFs such as phosphatidylinositol 3,4,5-triphosphate-dependent Rac exchanger 1 (P-Rex1), T-cell lymphoma invasion and metastasis 1 (Tiam1), and vav guanine nucleotide exchange factor 3 (Vav3) have been also implicated in breast cancer invasion and metastasis (reviewed in Wertheimer et al. [2]). However, the clinical and/or biological significance of Rho/Rac GAPs has remained largely unclear.

Rho GTPase avtivating protein 15 (ARHGAP15) is a recently identified Rac GAP and is specific for Rac1 [8]. Previously, we identified *ARHGAP15* as an androgen-induced gene by microarray analysis [9]. Recently, ARHGAP15 has been reported to be expressed in human gliomas and to suppress migration and invasion of U87 and U251 human glioma cells [10]. However, ARHGAP15 and its regulation have not been examined in breast carcinomas. Therefore, we examined the significance of ARHGAP15 using immunohistochemistry and explored the effects of ARHGAP15 on breast cancer cell proliferation and migration. Furthermore, we examined possible regulation of ARHGAP15 expression by androgens.

## 2. Results

### 2.1. Immunolocalization of ARHGAP15 and Rac1 in Human Breast Carcinoma Tissues

**Figure 1:**
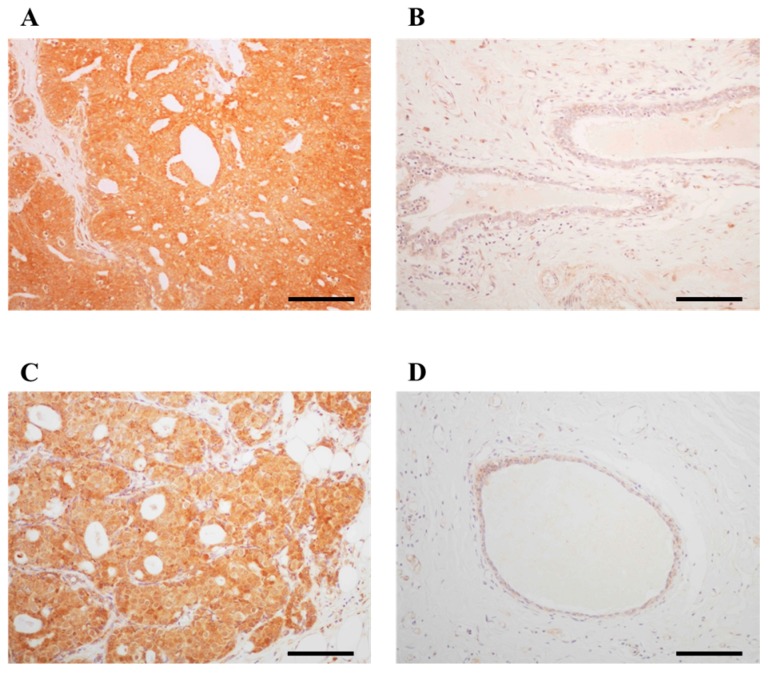
Immunolocalization of ARHGAP15 and Rac1 in human breast carcinoma. Immunoreactivity of ARHGAP15 and Rac1 was detected in the cytoplasm of carcinoma cells (**A**,**C**), while almost negligible in non-neoplastic mammary epithelium (**B**,**D**). Scale bar is 100 μm for all figures.

ARHGAP15 immunoreactivity was detected in the cytoplasm of carcinoma cells (Figure 1A). On the other hand, it was almost negligible in non-neoplastic mammary epithelium and only slightly detected in the stroma (Figure 1B). Rac1 immunoreactivity was detected in the cytoplasm of carcinoma cells (Figure 1C), while it was almost negligible in non-neoplastic mammary epithelium or stroma (Figure 1D). The association between clinicopathological parameters and ARHGAP15 and Rac1 is summarized in Table 1 and Table 2, respectively. Significant correlation was detected between ARHGAP15 immunoreactivity and androgen receptor (AR) labeling index (LI) (*p* = 0.033). Interestingly, ARHGAP15 immunoreactivity was also positively correlated with that of Rac1 (*p* = 0.003). On the other hand, Rac1 immunoreactivity negatively correlated with estrogen receptor (ER) (*p* = 0.022) and progesterone receptor (PR) status (*p* = 0.0044). Although the *p* value did not reach a significant level, Rac1 immunoreactivity was positively associated with lymph node metastasis (*p* = 0.088).

### 2.2. Correlation between ARHGAP15, Rac1 Immunoreactivity, and Clinical Outcome of Breast Carcinoma Patients

**Figure 2:**
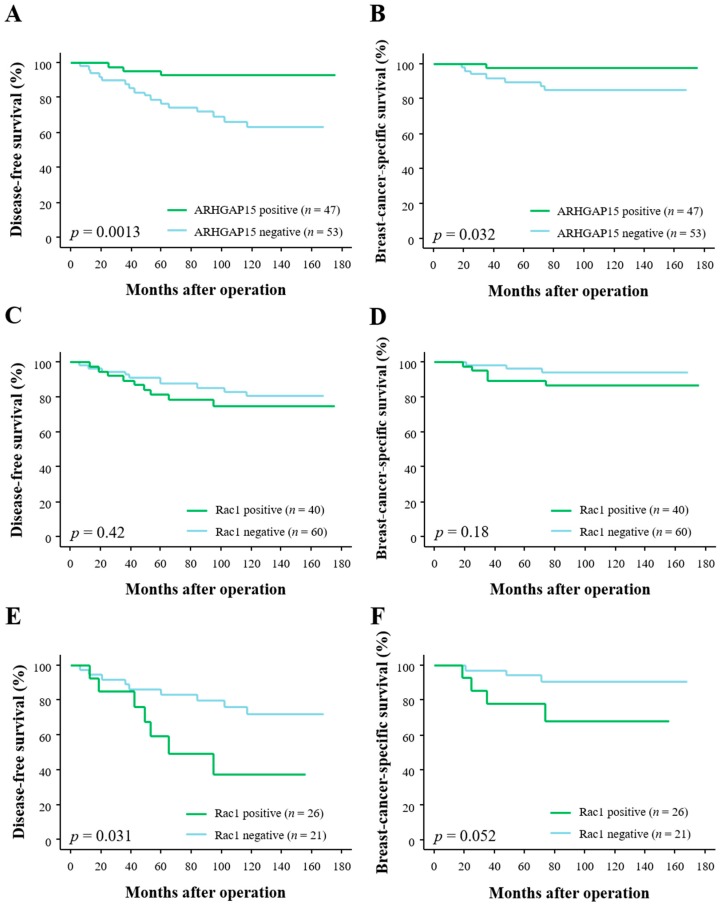
Disease-free survival and breast-cancer-specific survival of 100 breast carcinoma patients according to ARHGAP15 and Rac1 immunoreactivity. (**A**,**B**) Disease-free survival (**A**) and breast-cancer-specific survival (**B**) according to ARHGAP15 immunoreactivity. (**C**,**D**) Disease-free survival (**C**) and breast-cancer-specific survival (**D**) according to Rac1. (**E**,**F**) Disease-free survival (**E**) and breast-cancer-specific survival (**F**) according to Rac1 in ARHGAP15-negative cases (*n* = 47).

In order to address the prognostic implications of ARHGAP15 and Rac1 in human breast carcinoma patients, we associated the immunoreactivity of them with the clinical outcome of the patients. As shown in Figure 2A, ARHGAP15 immunoreactivity was significantly associated with decreased risk of recurrence (*p* = 0.0013), and multivariate analyses demonstrated that ARHGAP15, lymph node metastasis, and human epidermal growth factor receptor 2 (HER2) status were independent prognostic factors for disease-free survival with relative risk over 1.0 (Table 3).

Correlation between ARHGAP15 immunoreactivity and breast-cancer-specific survival is summarized in Figure 2B, and ARHGAP15 immunoreactivity was significantly associated with improved clinical outcome (*p* = 0.032). Subsequent multivariate analyses demonstrated that ARHGAP15 as well as histological grade and HER2 status were independent prognostic factors for clinical outcome with relative risk of over 1.0 Table 4).

When we associated Rac1 immunoreactivity with disease-free survival and breast-cancer-specific survival, we could not detect any statistically significant difference between Rac1-positive and Rac1-negative cases (*p* = 0.42 for disease-free survival (Figure 2C) and *p* = 0.18 for breast-cancer-specific survival (Figure 2D)). However, when we further analyzed according to ARHGAP15 status, Rac1 immunoreactivity was significantly associated with increased risk of recurrence in ARHGAP15-negative cases (*p* = 0.031, Figure 2E). Although the *p* value did not reach significant level, Rac1 immunoreactivity tended to be associated with adverse clinical outcome in ARHGAP15-negative cases (*p* = 0.052, Figure 2F).

### 2.3. Effects of ARHGAP15 on Rac1 Activation, Proliferation, and Migration of Breast Carcinoma Cells

**Figure 3:**
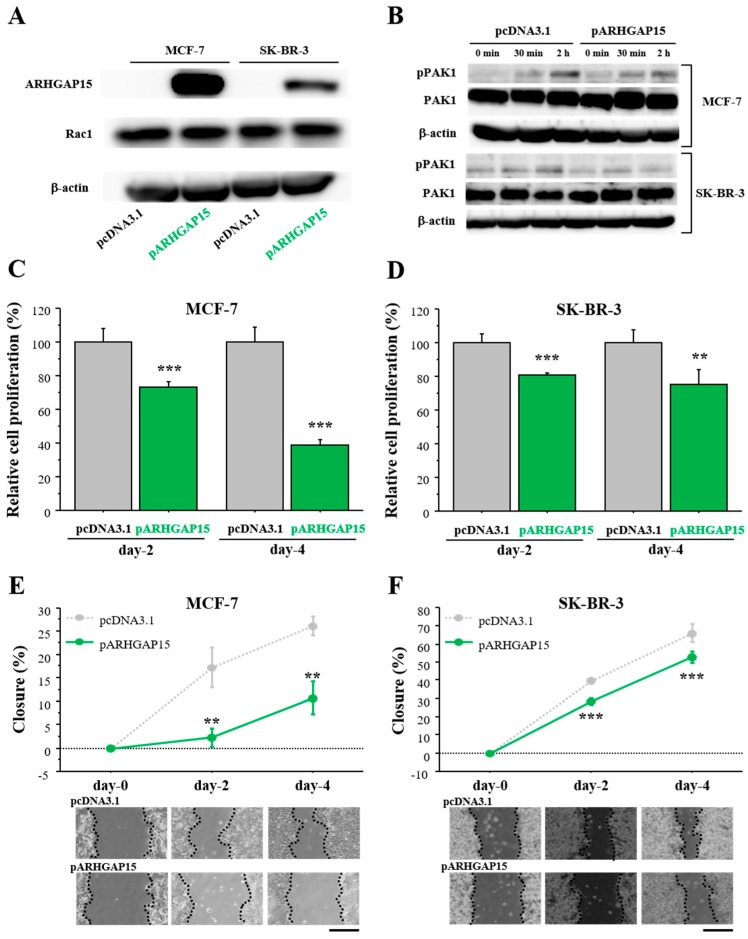
Effects of ARHGAP15 on Rac1 activation, proliferation, and migration of breast carcinoma cells. (**A**) Expression of ARHGAP15 protein in MCF-7 cells and SK-BR-3 cells transfected with ARHGAP15 expression vector (pARHGAP15) or vacant vector (pcDNA3.1). (**B**) MCF-7 (upper panel) and SK-BR-3 cells (lower panel) were transfected with ARHGAP15 expression vector or vacant vector, followed by stimulation with HRG (10 ng/mL) for the indicated time in serum-free conditions. (**C**,**D**) Relative cell proliferation of MCF-7 cells (**C**) and SK-BR-3 cells (**D**) transfected with pARHGAP15 was summarized as the ratio (%) to those transfected with vacant vector (2 days and 4 days after transfection). (**E**,**F**) Wound healing assays in MCF-7 cells (**E**) and SK-BR-3 cells (**F**). Relative migration area was evaluated as the ratio (%) to those at the removal of culture inserts (0 h). Scale bar is 500 μm. Data were presented as mean ± standard deviation (SD). ** *p* < 0.01 and *** *p* < 0.001. Lower panels show representative micrographs in each period. PAK1: p21 (RAC1)-activated kinase 1.

In order to examine the effects of ARHGAP15 on breast cancer progression, we performed cell proliferation assay and migration assay. At first, we confirmed that MCF-7 and SK-BR-3 cells did not express a significant level of ARHGAP15 protein, but expressed abundant Rac1 protein (Figure 3A). Therefore, we constructed an ARHGAP15 expression vector and transfected it into MCF-7 cells or SK-BR-3 cells. As shown in Figure 3A, the ARHGAP15 protein was markedly increased by the transfection of the ARHGAP15 expressing vector, while the RAC1 protein level was not changed. Next, we examined whether exogenous ARHGAP15 expression caused suppression of Rac1 activation. MCF-7 and SK-BR-3 cells were transfected with the ARHGAP15 expressing vector, followed by stimulation with heregulin-β1 (HRG; 10 ng/mL). HRG is known to induce Rac1 activation and subsequent phosphorylation of p21 (RAC1)-activated kinase 1 (PAK1), a critical effector protein of Rac1 signaling, in breast cancer cells [11]. As shown in Figure 3B, HRG-induced phosphorylation of PAK1 was suppressed when MCF-7 and SK-BR-3 cells were transfected with the ARHGAP15 expression vector. The effects of ARHGAP15 on breast cancer cell proliferation are summarized in Figure 3C (MCF-7) and Figure 3D (SK-BR-3). Cell proliferation was significantly suppressed in MCF-7 transfected with the ARHGAP15 expressing vector (Day 2: 0.74-fold and *p* < 0.001; Day 4: 0.39-fold and *p* < 0.001). A similar tendency was also observed in SK-BR-3 cells (Day 2: 0.81-fold and *p* < 0.001; Day 4: 0.75-fold and *p* < 0.01).

Cell migration was examined by wound healing assay using MCF-7 cells (Figure 3E) and SK-BR-3 cells (Figure 3F). Relative cell migration was significantly suppressed in both MCF-7 cells (Day 2: 0.13-fold and *p* < 0.01; Day 4: 0.41-fold and *p* < 0.01) and SK-BR-3 cells (Day 2: 0.72-fold and *p* < 0.001; Day 4: 0.80-fold and *p* < 0.001) transfected with the ARHGAP15 expression vector. 

### 2.4. Regulation of ARHGAP15 and RAC1 Expression by Sex Steroids in Human Breast Carcinomas

**Figure 4:**
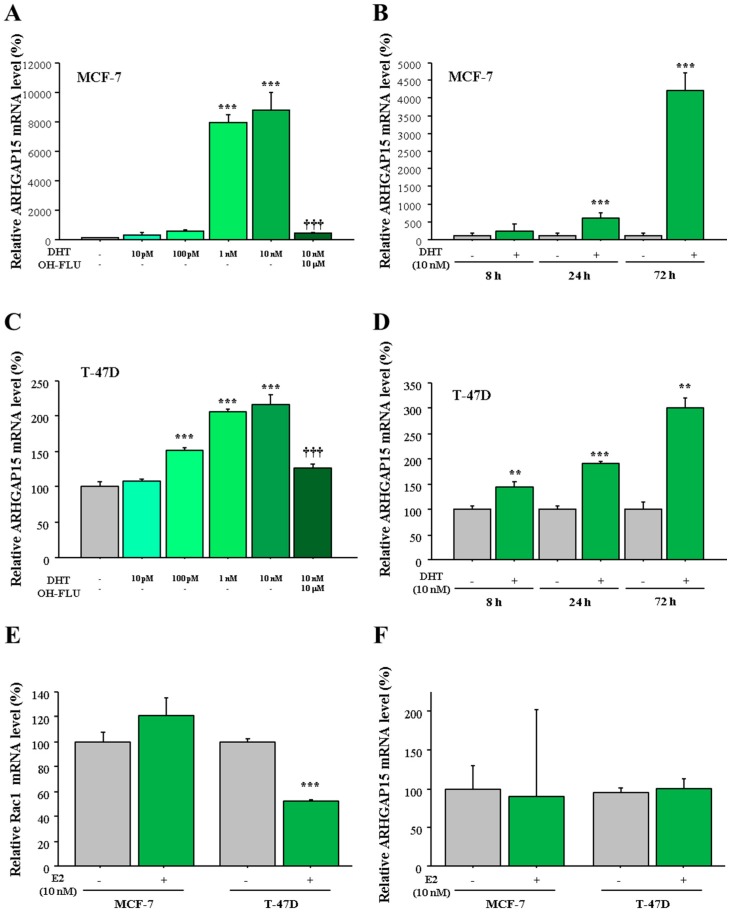
Regulation of *ARHGAP15* mRNA by DHT in MCF-7 cells and T-47D cells. (**A**,**C**) MCF-7 cells (**A**) and T-47D cells (**C**) were treated with the indicated concentration of DHT for 72 h with or without hydroxyflutamide (OH-FLU), and expression of *ARHGAP15* mRNA was examined using real-time PCR. *ARHGAP15* mRNA was evaluated as the ratio of *RPL13A* mRNA and subsequently the relative *ARHGAP15* mRNA level was summarized as the ratio (%) to basal level (nontreatment). (**B**,**D**) MCF-7cells (**B**) and T-47D cells (**D**) were treated with 10 nM DHT for indicated periods, and relative *ARHGAP15* mRNA level was summarized as the ratio (%) to the basal level of each period. (**E**,**F**) MCF-7 and T-47D cells were treated with 10 nM estradiol (E2) for 72 h and relative *Rac1* mRNA level (**E**) and *ARHGAP15* mRNA level (**F**) were summarized as the ratio to basal level. Data were presented as mean ± standard deviation (SD). ** *p* < 0.01, *** *p* < 0.001 compared to basal level and ^†††^ *p* < 0.001 compared to DHT-treated group (10 nM).

In the histochemical analyses, we detected significant positive correlation between ARHGAP15 immunoreactivity and AR LI. On the other hand, we detected significant negative correlation between RAC1 immunoreactivity and both ER and PR status. In addition, we previously reported that *ARHGAP15* mRNA was increased by DHT, as discovered during microarray experiments using T-47D cells [9]. Therefore, we examined the possible regulation of ARHGAP15 and RAC1 by sex steroids using MCF-7 and T-47D cells which expressed AR as well as ER [12]. As shown in Figure 4A, expression of *ARHGAP15* mRNA was significantly induced by DHT in a dose-dependent fashion (88-fold and *p* < 0.001 at 10 nM DHT) in MCF-7 cells, which was significantly suppressed in the presence of the potent AR inhibitor hydroxyflutamide (OH-FLU). The induction of *ARHGAP15* mRNA by DHT was observed time-dependently, and became significant from 24 h (5.9-fold and *p* < 0.001, Figure 4B). A similar tendency was also observed when T-47D cells were used (Figure 4C,D).

On the other hand, *Rac1* mRNA was significantly suppressed by estradiol (E2, 10 nM, 0.53-fold and *p* < 0.001) in T-47D cells, while it was not induced by E2 in MCF-7 cells (*p* = 0.10, Figure 4E). In addition, *ARHGAP15* mRNA expression was not affected by E2 in MCF-7 and T-47D cells (Figure 4F).

## 3. Discussion

To the best of our knowledge, this is the first study which has examined the clinical significance of ARHGAP15 in human breast carcinomas. In the present study, ARHGAP15 immunoreactivity was detected in 47% of breast carcinoma cases, and was significantly associated with that of Rac1, while it was almost negligible in morphologically normal mammary epithelium. On the other hand, the importance of ARHGAP15 in the progression of human gliomas has been also reported [11]. Therefore, it is suggested that ARHGAP15 may be expressed and have pivotal roles in the progress of human malignancies including breast carcinomas, affecting the Rac1 pathway.

Our study demonstrated that ARHGAP15 immunoreactivity was significantly associated with decreased risk of recurrence and better prognosis in breast carcinoma patients. ARHGAP15 serves as a Rac GAP protein and inactivates Rac1 activity by enhancing the intrinsic GTPase activity of Rac1 [8]; we also demonstrated that exogenous expression of ARHGAP15 resulted in suppression of HRG-dependent activation of Rac1. Furthermore, Rac1 has been reported as an unfavorable prognostic factor in breast carcinomas [13]. Actually, Rac1 immunoreactivity was significantly associated with shorter disease-free survival in the cases negative for ARHGAP15 in our present study. It was therefore reasonably considered that ARHGAP15 may suppress the progress of breast carcinomas by attenuating the Rac1 pathway and may serve as better prognostic factor in breast carcinomas. However, it is also true that some Rac GAP such as P190B or Rac GTPase activating protein (RACGAP1) have pro-tumorigenic function [14,15,16]. Rac GAP may therefore have diverse functions and further examinations are needed.

In the present study, exogenous overexpression of ARHGAP15 resulted in a significant decrease of cell migration in MCF-7 and SK-BR-3 cells. These results are in good agreement with previous reports by Sun et al. [10], which demonstrated knockdown of ARHGAP15 suppressed migration and invasion of U87 and U258 glioma cells. It has also been reported that Rac1 stimulates migration and invasion of breast cancer cells [17,18]. ARHGAP15 is therefore considered to suppress migration of breast carcinoma cells by inactivating Rac1.

In addition, cell proliferation was also suppressed by exogenous ARHGAP15 overexpression in MCF-7 and SK-BR-3 cells. In addition, Rac1 has been reported to be associated with proliferation of cancer cells, partially by regulating cell cycle progression [19,20,21,22,23]. These findings indicated possible antiproliferative effects of ARHGAP15. However, it is also true that Rac1 immunoreactivity is not associated with Ki67 LI, which well reflects proliferative activity of breast carcinoma cells [24]. This discrepancy may be partly due to the fact that the antibody for Rac1 used in this study recognizes both the GTP-bound form and the GDP-bound form, and immunoreactivity does not always reflect the activity of Rac1. We therefore examined the correlation between Rac1 and Ki67 LI in the cases negative for ARHGAP15, but we could not detect a significant correlation between them. Further examinations with a larger number of samples may be needed in order to clarify more precisely the roles of ARHGAP15 in breast cancer cell proliferation.

In our previous study, we pointed out that expression of *ARHGAP15* mRNA is possibly regulated by androgens in breast cancer cells after results found using microarray analysis [9]. Consistent with this previous study, we detected a positive correlation between ARHGAP15 immunoreactivity and AR LI. We subsequently examined possible regulation of ARHGAP15 by androgens by using real-time PCR which revealed that *ARHGAP15* mRNA was induced by DHT in both time- and dose-dependent fashions in MCF-7 cells and T-47D cells. We also confirmed that this induction was markedly suppressed by the addition of OH-FLU, a potent AR inhibitor. Therefore, ARHGAP15 is considered an androgen-induced gene in human breast carcinomas. Although some inconsistent findings have been reported, androgens are considered to exert anti-tumorigenic effects on breast cancer (reviewed in Suzuki et al. [25] and Takagi et al. [26]). Considering that ARHGAP15 suppresses breast cancer cell proliferation and migration as described above, it may be possible to speculate that anti-tumorigenic effects of androgens are partially due to the induction of ARHGAP15 and subsequent inactivation of Rac1. On the other hand, ARHGAP15 is reported to be regulated by transcription factor forkhead box P3 (FOXP3) in glioma cells [10]. Although FOXP3 is well known to be expressed in regulatory T cells and associated with their differentiation and function, recent studies have explored the hypothesis that FOXP3 is expressed not only in normal breast epithelium and also in breast carcinoma cells [27,28]. Expression of ARHGAP15 is therefore considered to be regulated by several mechanisms other than androgens, such as FOXP3, in breast carcinomas.

On the other hand, we found that Rac1 immunoreactivity was negatively correlated with ER and PR, and *Rac1* mRNA was significantly suppressed in T-47D cells by E2—biologically active estrogens. This may be consistent with a previous study which reported that Rac1 expression was down-regulated by estrogens in vascular smooth muscle cells [29]. It is well known that estradiol is locally produced by estrogen-producing enzymes such as aromatase [30]. Expression of Rac1 may be partly regulated by locally produced estrogens in breast carcinomas. It is therefore hypothesized that Rac1 activity may be influenced by intratumoral sex steroids in breast carcinomas. However, it is also true that suppression of *Rac1* mRNA by E2 was not observed in MCF-7 cells, suggesting the possibility that regulation of Rac1 expression by estrogens is cell-type-specific. It may be helpful to address this hypothesis for better understanding of sex steroid dependency of breast carcinomas.

In summary, we immunolocalized ARHGAP15 and Rac GAP in human breast carcinoma tissues, and ARHGAP15 immunoreactivity was significantly correlated with AR LI, decreased risk of recurrence, and better prognosis for the patients. Subsequent in vitro experiments revealed that ARHGAP15 suppressed cell proliferation and migration in MCF-7 cells and SK-BR-3 cells. In addition, expression of ARHGAP15 was possibly regulated by androgens. These results suggested that ARHGAP15 is an androgen-induced gene and has anti-tumorigenic roles by way of inactivating the Rac1 pathway in breast carcinomas.

## 4. Materials and Methods

### 4.1. Patients and Tissues

One hundred specimens of invasive ductal carcinoma of the breast were obtained from female patients who underwent surgical treatment from 1990 to 1999 at Tohoku University Hospital, Sendai, Japan. No patients received any neoadjuvant therapies, including chemotherapy, irradiation, or endocrine therapy. Clinical outcome of the patients was evaluated by disease-free survival and breast-cancer-specific survival. The mean age of the patients was 57.2 years (range 31–81 years), and the mean follow-up period was 106 months (range 1–175 months). Ten percent formalin-fixed paraffin-embedded tissues were used for immunohistochemistry. This study was approved by the Ethics Committee at Tohoku University School of Medicine (approval No. 2015-1-162, 15 July 2015).

### 4.2. Immunohistochemistry

Mouse polyclonal antibody for ARHGAP15 (ab69068) was purchased from Abcam (Cambridge, UK). Rabbit polyclonal antibodies for Rac1 (ab78139) and HER2 (A0485) were purchased from Abcam and DAKO (Carpinteria, CA, USA), respectively.

Mouse monoclonal antibodies for ER (ER1D5), PR (MAB429), AR (AR441) and Ki67 (MIB1) were purchased from DAKO. A Histofine kit (Nichirei, Tokyo, Japan) was used for detecting antigen–antibody complexes, visualized with 3,3′-diaminobenzidine and counterstained by hematoxylin. Human kidney was used as a positive control for ARHGAP15. As a negative control, PBS was used instead of the primary antibody.

### 4.3. Scoring of Immunoreactivity

Immunoreactivity of ARHGAP15 and Rac1 was detected in the cytoplasm of carcinoma cells. Immunoreactivity of more than 10% of carcinoma cells was defined as positive for ARHGAP15 and Rac1. Immunoreactivity of ER, PR, and AR was detected in the nuclei of carcinoma cells, and percentage of immunoreactivity (labeling index; LI) was determined by counting more than 1000 cells. Cases with ER or PR LI of more than 1% were defined as positive for ER or PR [31]. HER2 immunoreactivity was evaluated according to the standardized HercepTest scoring system (DAKO), and a score of 3+ was defined as positive.

### 4.4. Cell Lines and Chemicals

Human breast cancer cell lines MCF-7, SK-BR-3 and T-47D were obtained from Japanese Collection of Research Bioresources Cell Bank (Osaka, Japan) and American Type Culture Collection (ATCC; Manassas, VA, USA), respectively. MCF-7 cells and T-47D cells were cultured in RPMI-1640 (Wako, Osaka, Japan). SK-BR-3 cells were cultured in McCoy’s 5A (Gibco, Rockville, MD, USA). Heregulin-β1 was purchased from Wako.

### 4.5. Real-Time RT-PCR

TRI reagent (Molecular Research Center, Cincinnati, OH, USA) was used for the extraction of total RNA from cell lines, and cDNA was synthesized using ReverTra Ace qPCR RT Master Mix (TOYOBO, Osaka, Japan). Real-time PCR was performed using THUNDERBIRD SYBR qPCR Mix (TOYOBO). Sequences of primers used in this study were as follows: *ARHGAP15*, 5′-CCAAATCGAGACACCATGAA-3′ (forward) and 5′-GCAGAAGGGTAGGTCCAAAT-3′ (reverse); *Rac1*, 5′-CCTGGAGAATATATCCCTACTGTC-3′ (forward) and 5′-GGGGCGTAATCTGTCATAATCT-3′ (reverse); and *RPL13A*, 5′-CCTGGAGGAGAAGAGGAAAGAGA-3′ (forward) and 5′-TTGAGGACCTCTGTGTATTTGTCAA-3′ (reverse). *ARHGAP15* and *Rac1* mRNA levels were calculated as the ratio of *RPL13A* mRNA level (%).

### 4.6. Immunoblotting

Total protein was extracted using M-PER Mammalian Protein Extraction Reagent (Pierce Biotechnology, Rockford, IL, USA) with Protease Inhibitor Cocktail (Sigma-Aldrich, St. Louis, MO, USA). A quantity of 20 g of protein was subjected to sodium dodecyl sulfate-polyacrylamide gel electrophoresis (SDS-PAGE), and transferred onto a Hybond PVDF membrane (GE Healthcare, Buckinghamshire, UK). The primary antibody for ARHGAP15 was the same as that used in immunohistochemistry. Rabbit polyclonal antibodies for PAK1 and phosphorylated PAK1/2 were purchased from Cell Signaling Technology (Danvers, MA, USA). Anti-b-actin antibody (A3854, Sigma-Aldrich) was used for equal loading control. Antibody–protein complexes on the membrane were detected using ECL-prime Western blotting detection reagents (GE Healthcare), and visualized by an LAS-4000 image analyzer (Fuji Photo Film Co., Tokyo, Japan).

### 4.7. Plasmid Construction and Transfection

ARHGAP15 expression plasmid was constructed by inserting a full-length open reading frame of human ARHGAP15 into pcDNA3.1 (−) vector (Invitrogen Life Technologies Inc., Carlsbad, CA, USA). The plasmid was transfected with Avalanche^®^-Everyday Transfection Reagent (APRO Science, Tokushima, Japan).

### 4.8. Cell Proliferation Assay and Wound Healing Assay

MCF-7 and SK-BR-3 cells were transfected with ARHGAP15 expression vector in 96-well culture plates, and subsequent cell proliferation was evaluated using a Cell Counting Kit-8 (Dojindo, Molecular Technologies, Kumamoto, Japan).

The cell migration property of breast cancer cells was evaluated by wound healing assay. MCF-7 and SK-BR-3 cells were seeded in Culture-Inserts (Ibidi, GmbH, Munich, Germany) at >90% confluency. After cell adherence, the Culture-Inserts were removed and remaining gaps were quantified using NIS Elements software v3.0 (Nikon, Tokyo, Japan).

### 4.9. Statistical Analyses

JMP pro 13.1.0 (SAS Institute, Cary, NC, USA) was used for statistical analyses. Correlation between ARHGAP15 and/or Rac1 immunoreactivity and clinicopathological parameters was examined using Mann–Whitney’s *U* test or *χ*^2^ test. The Kaplan–Meier method and log-rank test were employed to evaluate the prognostic significance of ARHGAP15 and Rac1. Univariate and multivariate analyses were performed using a proportional hazard model (Cox). Dunnett’s test was used for the evaluation of statistical significance in all in vitro experiments.

## Figures and Tables

**Table 1:** Association between ARHGAP15 immunoreactivity and clinicopathological parameters in breast carcinoma patients (*n* = 100).

	ARHGAP15 Immunoreactivity		*p* Value
	Negative (*n* = 53)	Positive (*n* = 47)	
Age	59 (36–81)	56 (31–80)	0.65
Menopausal status			
Premenopausal	16	14	
Postmenopausal	37	33	0.97
Stage			
I	17	17	
II	27	23	
III	9	7	0.90
pT			
pT1	21	21	
pT2–4	32	26	0.61
Lymph node metastasis			
Negative	31	30	
Positive	22	17	0.58
Histological grade			
1 (well)	9	15	
2 (intermediate)	26	21	
3 (poor)	18	11	0.19
ER status			
Negative	9	10	
Positive	44	37	0.59
PR status			
Negative	13	14	
Positive	40	33	0.55
AR LI (%) *	14.5 (0–86)	28 (0–89)	**0.033**
HER2 status			
Negative	45	37	
Positive	8	10	0.42
Ki-67 LI (%) *	17 (0–82)	14 (0–54)	0.42
Rac1			
Negative	39	21	
Positive	14	26	**0.003**

* Data was presented as median (minimum – maximum) and statistical analyses were performed using Mann–Whitney’s *U* test. All other values were presented as the number of cases and statistical analyses were performed using *χ*^2^ test. A *p* value less than 0.05 was considered significant and shown in bold. AR: androgen receptor; ER: estrogen receptor; HER2: human epidermal growth factor receptor 2; PR: progesterone receptor; pT: pathological T factor.

**Table 2:** Association between Rac1 immunoreactivity and clinicopathological parameters in breast carcinoma patients (*n* = 100).

	Rac1 Immunoreactivity		*p* Value
	Negative (*n* = 60)	Positive (*n* = 40)	
Age	56.5 (31–81)	59 (37–80)	0.33
Menopausal status			
Premenopausal	22	8	
Postmenopausal	38	32	0.074
Stage			
I	23	11	
II	30	20	
III	7	9	0.27
pT			
pT1	29	13	
pT2–4	31	27	0.12
Lymph node metastasis			
Negative	39	22	
Positive	21	18	0.088
Histological grade			
1 (well)	14	10	
2 (intermediate)	33	14	
3 (poor)	13	16	0.19
ER status			
Negative	7	12	
Positive	53	28	**0.022**
PR status			
Negative	10	17	
Positive	50	23	**0.0044**
AR LI (%) *	21.5 (0–89)	17 (0–83)	0.87
HER2 status			
Negative	51	31	
Positive	9	9	0.34
Ki-67 LI (%) *	14 (0–73)	18.5 (0–82)	0.14

* Data was presented as median (minimum – maximum) and statistical analyses were performed using Mann–Whitney’s *U* test. All other values were presented as the number of cases and statistical analyses were performed using *χ*^2^ test. A *p* value less than 0.05 was considered significant and shown in bold.

**Table 3:** Univariate and multivariate analyses of disease-free survival in breast carcinoma patients (*n* = 100).

Parameter	Univariate	Multivariate	
	*p* Value	*p* Value	Relative Risk (95% CI)
Age (31–81) *	0.77		
Menopausal status (Post-/Pre-)	0.62		
pT (2–4/1)	*0.077*	0.17	
Lymph node metastasis (positive/negative)	**0.025**	**0.0087**	**3.8 (1.4–12)**
Histological grade (3/1,2)	0.89		
ER status (negative/positive)	0.89		
PR status (negative/positive)	0.26		
AR status (negative/positive)	0.47		
HER2 status (positive/negative)	*0.089*	**0.0085**	**4.7 (1.5–14)**
Ki67 (≥10/<10)	0.60		
ARHGAP15 (negative/positive)	**0.0008**	**0.0005**	**6.9 (2.2–31)**
Rac1 (positive/negative)	0.43		

Statistical analysis was performed using a proportional hazard model (Cox). Values *p* < 0.05 and 0.05 ≤ *p* ≤ 0.1 were considered significant (bold) and borderline significant (italic), respectively, and were examined in multivariate analysis. * Data was used as a continuous variable, and all other parameters were used as dichotomized variables. 95% CI: 95% confidence interval.

**Table 4:** Univariate and multivariate analyses of breast-cancer-specific survival in breast carcinoma patients (*n* = 100).

Parameter	Univariate	Multivariate	
	*p* Value	*p* Value	Relative Risk (95% CI)
Age (31–81) *	0.88		
Menopausal status (Post-/Pre-)	0.72		
pT (2–4/1)	*0.053*	0.37	
Lymph node metastasis (positive/negative)	0.17		
Histological grade (3/1,2)	**0.0061**	**0.042**	**4.8 (1.06–35)**
ER status (negative/positive)	0.18		
PR status (negative/positive)	0.45		
AR status (negative/positive)	0.34		
HER2 status (positive/negative)	**0.039**	**0.043**	**4.6 (1.1–20)**
Ki67 (≥10/<10)	0.13		
ARHGAP15 (negative/positive)	**0.023**	**0.016**	**8.5 (1.4–163)**
Rac1 (positive/negative)	0.19		

Statistical analysis was performed using a proportional hazard model (Cox). Values *p* < 0.05 and 0.05 ≤ *p* ≤ 0.1 were considered significant (bold) and borderline significant (italic), respectively, and were examined in multivariate analysis. * Data was used as a continuous variable, and all other parameters were used as dichotomized variables. 95% CI: 95% confidence interval.

## Abbreviations

AR	Androgen receptor
ARHGAP15	Rho GTPase activating protein
DHT	Dihydrotestosterone
Cdc42	Cell division cycle 42
ER	Estrogen receptor
E2	Estradiol
HER2	Human epidermal growth factor receptor 2
HRG	Heregulin-β1
LI	Labeling index
OH-FLU	Hydroxyflutamide
PAK1	Phosphorylation of p21 (RAC)-activated kinase 1
PR	Progesterone receptor
P-Rex1	phosphatidylinositol 3,4,5-triphosphate-dependent Rac exchanger 1
pT	Pathological T factor
Rac1	Ras-related C3 botulinum toxin substrate 1
Rho	Ras-related homolog
RhoBTB	Rho related BTB domain containing
Tiam1	T-cell lymphoma invasion and metastasis 1
Wrch	Wnt responsive Cdc42 homolog
Vav3	vav guanine nucleotide exchange factor 3

## References

[1] Manser E. (2002). Small GTPases take the stage. Dev. Cell.

[2] Wertheimer E., Gutierrez-Uzquiza A., Rosemblit C., Lopez-Haber C., Sosa M.S., Kazanietz M.G. (2012). Rac signaling in breast cancer: A tale of GEFs and GAPs. Cell. Signal..

[3] Jansen S., Gosens R., Wieland T., Schmidt M. (2018). Paving the Rho in cancer metastasis: Rho GTPases and beyond. Pharmacol. Ther..

[4] Bustelo X.R., Sauzeau V., Berenjeno I.M. (2007). GTP-binding proteins of the Rho/Rac family: Regulation, effectors and functions in vivo. Bioessays.

[5] Dovas A., Couchman J.R. (2005). RhoGDI: Multiple functions in the regulation of Rho family GTPase activities. Biochem. J..

[6] Dokmanovic M., Hirsch D.S., Shen Y., Wu W.J. (2009). Rac1 contributes to trastuzumab resistance of breast cancer cells: Rac1 as a potential therapeutic target for the treatment of trastuzumab-resistant breast cancer. Mol. Cancer Ther..

[7] Johnson E., Seachrist D.D., DeLeon-Rodriguez C.M., Lozada K.L., Miedler J., Abdul-Karim F.W., Keri R.A. (2010). HER2/ErbB2-induced breast cancer cell migration and invasion require p120 catenin activation of Rac1 and Cdc42. J. Biol. Chem..

[8] Seoh M.L., Ng C.H., Yong J., Lim L., Leung T. (2003). ArhGAP15, a novel human RacGAP protein with GTPase binding property. FEBS Lett..

[9] Takagi K., Miki Y., Nagasaki S., Hirakawa H., Onodera Y., Akahira J., Ishida T., Watanabe M., Kimijima I., Hayashi S. (2010). Increased intratumoral androgens in human breast carcinoma following aromatase inhibitor exemestane treatment. Endocr. Relat. Cancer.

[10] Sun Z., Zhang B., Wang C., Fu T., Li L., Wu Q., Cai Y., Wang J. (2017). Forkhead box P3 regulates ARHGAP15 expression and affects migration of glioma cells through the Rac1 signaling pathway. Cancer Sci..

[11] Sosa M.S., Lopez-Haber C., Yang C., Wang H., Lemmon M.A., Busillo J.M., Luo J., Benovic J.L., Klein-Szanto A., Yagi H. (2010). Identification of the Rac-GEF P-Rex1 as an essential mediator of ErbB signaling in breast cancer. Mol. Cell.

[12] Migliaccio A., Castoria G., Di Domenico M., de Falco A., Bilancio A., Lombardi M., Barone M.V., Ametrano D., Zannini M.S., Abbondanza C. (2000). Steroid-induced androgen receptor-oestradiol receptor β-Src complex triggers prostate cancer cell proliferation. EMBO J..

[13] Liu B., Xiong J., Liu G., Wu J., Wen L., Zhang Q., Zhang C. (2017). High expression of Rac1 is correlated with partial reversed cell polarity and poor prognosis in invasive ductal carcinoma of the breast. Tumour Biol..

[14] McHenry P.R., Sears J.C., Herrick M.P., Chang P., Heckman-Stoddard B.M., Rybarczyk M., Chodosh L.A., Gunther E.J., Hilsenbeck S.G., Rosen J.M. (2010). P190B RhoGAP has pro-tumorigenic functions during MMTV-Neu mammary tumorigenesis and metastasis. Breast Cancer Res..

[15] Pliarchopoulou K., Kalogeras K.T., Kronenwett R., Wirtz R.M., Eleftheraki A.G., Batistatou A., Bobos M., Soupos N., Polychronidou G., Gogas H. (2013). Prognostic significance of *RACGAP1* mRNA expression in high-risk early breast cancer: A study in primary tumors of breast cancer patients participating in a randomized Hellenic Cooperative Oncology Group trial. Cancer Chemother. Pharmacol..

[16] Imaoka H., Toiyama Y., Saigusa S., Kawamura M., Kawamoto A., Okugawa Y., Hiro J., Tanaka K., Inoue Y., Mohri Y. (2015). RacGAP1 expression, increasing tumor malignant potential, as a predictive biomarker for lymph node metastasis and poor prognosis in colorectal cancer. Carcinogenesis.

[17] Keely P.J., Westwick J.K., Whitehead I.P., Der C.J., Parise L.V. (1997). Cdc42 and Rac1 induce integrin-mediated cell motility and invasiveness through PI(3)K. Nature.

[18] Zuo Y., Shields S.K., Chakraborty C. (2006). Enhanced intrinsic migration of aggressive breast cancer cells by inhibition of Rac1 GTPase. Biochem. Biophys. Res. Commun..

[19] Yamamoto M., Marui N., Sakai T., Morii N., Kozaki S., Ikai K., Imamura S., Narumiya S. (1993). ADP-ribosylation of the rhoA gene product by botulinum C3 exoenzyme causes Swiss 3T3 cells to accumulate in the G1 phase of the cell cycle. Oncogene.

[20] Olson M.F., Ashworth A., Hall A. (1995). An essential role for Rho, Rac, and Cdc42 GTPases in cell cycle progression through G1. Science.

[21] Moore K.A., Sethi R., Doanes A.M., Johnson T.M., Pracyk J.B., Kirby M., Irani K., Goldschmidt-Clermont P.J., Finkel T. (1997). Rac1 is required for cell proliferation and G2/M progression. Biochem. J..

[22] Yoshida T., Zhang Y., Rosado L.A.R., Chen J., Khan T., Moon S.Y., Zhang B. (2010). Blockade of Rac1 activity induces G1 cell cycle arrest or apoptosis in breast cancer cells through downregulation of cyclin D1, survivin, and X-linked inhibitor of apoptosis protein. Mol. Cancer Ther..

[23] Gastonguay A., Berg T., Hauser A.D., Schuld N., Lorimer E., Williams C.L. (2012). The role of Rac1 in the regulation of NF-κB activity, cell proliferation, and cell migration in non-small cell lung carcinoma. Cancer Biol. Ther..

[24] De Azambuja E., Cardoso F., de Castro G., Colozza M., Mano M.S., Durbecq V., Sotiriou C., Larsimont D., Piccart-Gebhart M.J., Paesmans M. (2007). Ki-67 as prognostic marker in early breast cancer: A meta-analysis of published studies involving 12,155 patients. Br. J. Cancer.

[25] Suzuki T., Miki Y., Takagi K., Hirakawa H., Moriya T., Ohuchi N., Sasano H. (2010). Androgens in human breast carcinoma. Med. Mol. Morphol..

[26] Takagi K., Miki Y., Ishida T., Sasano H., Suzuki T. (2017). The interplay of endocrine therapy, steroid pathways and therapeutic resistance: Importance of androgen in breast carcinoma. Mol. Cell. Endocrinol..

[27] Merlo A., Casalini P., Carcangiu M.L., Malventano C., Triulzi T., Mènard S., Tagliabue E., Balsari A. (2009). FOXP3 expression and overall survival in breast cancer. J. Clin. Oncol..

[28] Douglass S., Meeson A.P., Overbeck-Zubrzycka D., Brain J.G., Bennett M.R., Lamb C.A., Lennard T.W., Browell D., Ali S., Kirby J.A. (2014). Breast cancer metastasis: Demonstration that FOXP3 regulates CXCR4 expression and the response to CXCL12. J. Pathol..

[29] Laufs U., Adam O., Strehlow K., Wassmann S., Konkol C., Laufs K., Schmidt W., Böhm M., Nickenig G. (2003). Down-regulation of Rac-1 GTPase by Estrogen. J. Biol. Chem..

[30] Suzuki T., Miki Y., Nakamura Y., Moriya T., Ito K., Ohuchi N., Sasano H. (2005). Sex steroid-producing enzymes in human breast cancer. Endocr. Relat. Cancer.

[31] Hammond M.E., Hayes D.F., Dowsett M., Allred D.C., Hagerty K.L., Badve S., Fitzgibbons P.L., Francis G., Goldstein N.S., Hayes M. (2010). American Society of Clinical Oncology/College of American Pathologists guideline recommendations for immunohistochemical testing of estrogen and progesterone receptors in breast cancer. Arch. Pathol. Lab. Med..

